# Menstrual phase influences cerebrovascular responsiveness in females but may not affect sex differences

**DOI:** 10.3389/fphys.2022.1035452

**Published:** 2023-01-04

**Authors:** Bethany D. Skinner, Samuel R. C. Weaver, Samuel J. E. Lucas, Rebekah A. I. Lucas

**Affiliations:** ^1^ School of Sport, Exercise and Rehabilitation Sciences, University of Birmingham, Edgbaston, Birmingham, United Kingdom; ^2^ Centre for Human Brain Health, University of Birmingham, Edgbaston, Birmingham, United Kingdom

**Keywords:** cerebral blood flow, cerebrovascular function, female sex hormones, menstrual cycle, sex differences

## Abstract

**Background and aims:** Sex differences in the rate and occurrence of cerebrovascular diseases (e.g., stroke) indicate a role for female sex hormones (i.e., oestrogen and progesterone) in cerebrovascular function and regulation. However, it remains unclear how cerebrovascular function differs between the sexes, and between distinct phases of the menstrual cycle. This study aimed to compare cerebrovascular-CO_2_ responsiveness in 1) females during the early follicular (EF), ovulatory (O) and mid-luteal (ML) phases of their menstrual cycle; and 2) males compared to females during phases of lower oestrogen (EF) and higher oestrogen (O).

**Methods:** Eleven females (25 ± 5 years) complete experimental sessions in the EF (*n* = 11), O (*n* = 9) and ML (*n* = 11) phases of the menstrual cycle. Nine males (22 ± 3 years) completed two experimental sessions, approximately 2 weeks apart for comparison to females. Middle and posterior cerebral artery velocity (MCAv, PCAv) was measured at rest, during two stages of hypercapnia (2% and 5% CO_2_ inhalation) and hypocapnia (voluntary hyperventilation to an end-tidal CO_2_ of 30 and 24 mmHg). The linear slope of the cerebral blood velocity response to changes in end-tidal CO_2_ was calculated to measure cerebrovascular-CO_2_ responsiveness..

**Results:** In females, MCAv-CO_2_ responsiveness to hypocapnia was lower during EF (−.78 ± .45 cm/s/mmHg) when compared to the O phase (−1.17 ± .52 cm/s/mmHg; *p* < .05) and the ML phase (−1.30 ± .82; *p* < .05). MCAv-CO_2_ responsiveness to hypercapnia and hypo-to-hypercapnia, and PCAv-CO_2_ responsiveness across the CO_2_ range were similar between menstrual phases (*p* ≥ .20). MCAv-CO_2_ responsiveness to hypo-to hypercapnia was greater in females compared to males (3.12 ± .91 cm/s/mmHg vs. 2.31 ± .46 cm/s/mmHg; *p* = .03), irrespective of menstrual phase (EF or O).

**Conclusion:** Females during O and ML phases have an enhanced vasoconstrictive capacity of the MCA compared to the EF phase. Additionally, biological sex differences can influence cerebrovascular-CO_2_ responsiveness, dependent on the insonated vessel.

## 1 Introduction

Sex differences in the rate and occurrence of cerebrovascular diseases (e.g., stroke and vascular dementia) indicates a possible sex hormone specific role in brain vascular function and regulation. Increasing levels of oestrogen have been shown to lower cerebrovascular impedance (Krejza et al., 2004) and increase resting cerebral blood velocity (CB*v*; Krejza et al., 2001). Additionally, oestrogen has been reported to have neuroprotective effects, including suppression of the inflammatory response and increased perfusion after ischaemic injury (Hurn et al., 1995; Santizo et al., 2002). These oestrogen-related effects are thought to result from the enhanced production and activity of vasodilatory factors associated with prolonged oestrogen exposure (e.g., endothelial NO synthase, prostacyclin pathways; Krause et al., 2006). The effects of progesterone on the cerebrovasculature are less clear, with the literature suggesting it both promotes and reduces the inflammatory response (Gibson et al., 2005; Sunday et al., 2006). Of note, progesterone is believed to counteract the vasodilatory properties of oestrogen (Krause et al., 2006). Therefore, examination of both the vasodilatory and vasoconstrictive cerebrovascular response is needed to more fully understand regulation of the cerebrovasculature across the menstrual cycle. This broader range of assessment also has greater functional relevance (Hoiland et al., 2011). Manipulation of arterial CO_2_ content (either *via* inhalation of CO_2_ or *via* hyperventilation) allows for examination of both the vasodilatory (hypercapnic) and vasoconstrictive (hypocapnic) cerebrovascular response. Subsequently, cerebrovascular responsiveness to CO_2_ can be utilised to explore the possible opposing actions of oestrogen and progesterone.

Cyclic fluctuations in oestrogen and progesterone occur across the menstrual cycle, with these hormones at their lowest during the early follicular phase. Oestrogen peaks during ovulation, and a peak in progesterone occurs during the mid-luteal phase while oestrogen remains somewhat elevated. Despite the postulated neuroprotective effects of oestrogen, few studies have examined cerebrovascular function during distinct phases of the menstrual cycle. The vasodilatory capacity of the cerebrovasculature has been shown to be both similar between the early and late follicular phases (using TCD-derived CBv; Peltonen et al., 2016), and greater during the mid-luteal phase compared to the early and late follicular phases (using duplex Doppler-derived CBF; Krejza et al., 2013). As such, it is unclear how menstrual phase, and associated changes in female sex hormones, affects the vasoconstrictive capacity of the cerebrovasculature. Determining how cerebrovascular responsiveness across a broad CO_2_ range changes across the menstrual cycle will improve our understanding of the role of female sex hormones in both cerebrovascular health and disease.

Sex differences in CBv have been previously identified, with females displaying greater resting CBv than males (Oláh et al., 2000; Krejza et al., 2005). However, evidence on sex differences in cerebrovascular regulation is less clear, with cerebrovascular-CO_2_ responsiveness found to be greater in females compared to males (Kastrup et al., 1997; Oláh et al., 2000), similar between the sexes (Peltonen et al., 2015; Favre et al., 2020), and greater in males compared to females (Miller et al., 2019). Notably, these studies either did not control for the menstrual cycle or studied females only during the low hormone phase of the menstrual or oral contraceptive cycle, which may explain their contradictory findings. In addition, the majority of studies to date have only examined responses in the middle cerebral artery (MCA), yet regional differences in cerebrovascular-CO_2_ responsiveness have been reported (Sato et al., 2012; Skow et al., 2013). Subsequently, to characterise sex differences in cerebrovascular function more thoroughly, responses should be examined in more than one region where possible (e.g., anterior and posterior circulations). Overall, it is unclear how cerebrovascular responsiveness across a broad CO_2_ range, and in more than one vascular region, varies in males and females in different phases of the menstrual cycle.

Subsequently, this study aimed to compare cerebrovascular-CO_2_ responsiveness in 1) females during the early follicular (EF), ovulatory (O) and mid-luteal (ML) phases of their menstrual cycle; and 2) males compared to females during phases of lower oestrogen (EF) and higher oestrogen (O). Previous studies have shown oestrogen stimulates vasodilatory pathways, whereas progesterone and testosterone promote vasoconstriction. As such, it was hypothesised that cerebrovascular-CO_2_ responsiveness would be: 1) greater during the ovulatory phase when compared to the early follicular and mid-luteal phases, 2) lower in males compared to females in the ovulatory phase.

## 2 Materials and methods

The study was approved by the University of Birmingham Ethics Committee (ERN_15-1179) and all participants gave written, informed consent prior to enrolling in the study, in adherence with the Declaration of Helsinki.

### 2.1 Study design and protocol

**FIGURE 1:**
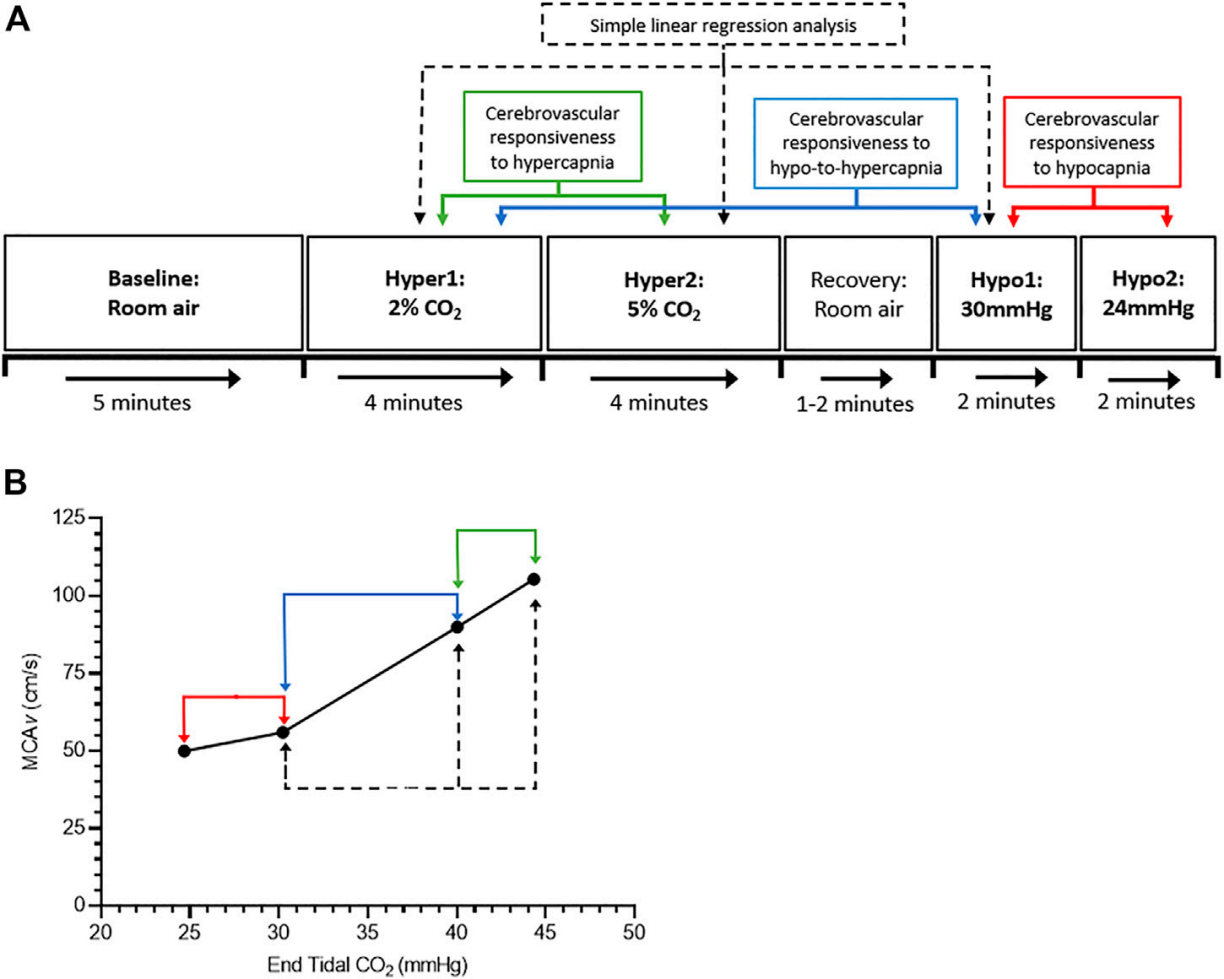
Schematic of the study protocol. **(A)** shows the cerebrovascular-CO_2_ responsiveness tests protocol, and **(B)** shows an example graph of the cerebrovascular-CO_2_ responsiveness slope. Green, blue, and red arrows indicate points of data extraction to calculate the cerebrovascular responsiveness to hypercapnia (Hyper1 to Hyper2), hypo-to-hypercapnia (Hypo1 to Hyper1), and hypocapnia (Hypo1 to Hypo2), respectively. Dashed back arrows indicate points of data extraction used in simple linear regression analysis.

Eleven females and 10 males participated in this study (see Table 1 for participant characteristics). Participants were required to attend the laboratory on either three (males) or four (females) occasions. During the first visit, written informed consent was obtained and a general health questionnaire completed to ensure inclusion/exclusion criteria were met. All participants were healthy and free of any known cardiovascular, neurological or metabolic diseases. Female participants had a regular menstrual cycle (<34 days in length) and were not taking any hormonal contraceptive medication. Following successful screening, participants completed a familiarisation session of the cerebrovascular-CO_2_ responsiveness protocol. They were asked to lie in a supine position for a minimum of 20 min prior to beginning the cerebrovascular-CO_2_ responsiveness tests (Figure 1). A 5 min period of resting data was collected with inhalation of room air, followed by two steps of hypercapnia (4 min each) and two steps of hypocapnia (∼2 min each). Hypercapnia was induced by inhalation of 2% and 5% CO_2_ gas mixtures (21% O_2_, balanced N_2_), and hypocapnia induced by guided voluntary hyperventilation to two end-tidal CO_2_ targets of 30 and 24 mmHg. The order of the cerebrovascular-CO_2_ responsiveness tests were kept consistent, as hypocapnia has been shown to blunt subsequent cerebrovascular responses to hypercapnia (Brothers et al., 2014).

**TABLE 1:** Participant characteristics.

	Females (*n = 11*)	Males (*n = 10*)
Age (years)	25 ± 5	22 ± 3
Height (m)	1.66 ± .05	1.78 ± .06^a^
Weight (kg)	64.1 ± 9.1	73.7 ± 4.9^a^
BMI (kg/m^2^)	23.2 ± 1.4	23.3 ± 1.5

^a^ Significantly different to females.

Female participants were asked to return for three experimental sessions during the early follicular, ovulatory and mid-luteal phases of their menstrual cycle. The order of visits was determined by which phase occurred first (see Supplementary Appendix SA1 for more details). An oral thermometer and calendar were provided with instructions to measure resting core temperature upon waking each morning prior to consumption of food or water to ensure accurate measurement. The early follicular phase was defined as days 1–4 of the menstrual cycle, where day 1 is the first day menstruation. Ovulation was defined as the 48-h period after a ≥.5°C increase in resting temperature, approximately halfway through the cycle. To confirm that participants were ovulating an ovulation test (Clearblue^®^) was performed following an increase in resting core temperature. The mid-luteal phase was ∼8–10 days post-ovulation, depending on total cycle length. Male participants were asked to return for two experimental sessions, approximately 2 weeks apart to replicate the time between the early follicular and ovulatory sessions for females. Upon arrival at an experimental testing session, participants were instrumented for measures of cerebro- and cardiovascular function (detailed below) before completing the cerebrovascular-CO_2_ responsiveness tests as described above.

### 2.2 Equipment and outcome measures

Beat-to-beat middle and posterior cerebral artery blood velocity (MCA*v*, PCA*v*) were assessed using transcranial Doppler (TCD; Doppler-Box X, DWL, Compumedics Ltd., Germany) with a 2-MHz probe placed over each temporal window. Ultrasound gel was placed on the probes and held in place with a headset. Where possible the right MCA and left PCA were insonated, though the PCA could not be positively identified in one female and one male participant.

Blood pressure was measured intermittently using an upper-arm cuff (Omron M5-I, Omron Healthcare Europe, Netherlands) and heart rate was collected beat-to-beat using a three-lead electrocardiogram. Respiratory rate and volume were measured using a heated pneumotachograph (3813 Series, Hans Rudolph Inc., United States) attached to a facemask, while fractional changes in inspired and expired O_2_ and CO_2_ were measured *via* a sample line attached to the facemask and a gas analyser (ML206, ADInstruments Ltd., New Zealand). Measures were recorded at 1 k Hz *via* an analogue-to-digital converter (Powerlab, ADInstruments) and displayed in real time and stored for offline analysis using commercially available software (LabChart v7.3.5, ADInstruments).

Circulating sex hormone concentrations (oestradiol, progesterone) were measured to verify menstrual phase status during each visit for female participants. Blood samples were drawn from the antecubital vein into 6 ml vacutainers containing K_2_EDTA as an anticoagulant (BD Vacutainer, United Kingdom). Samples were collected at rest following a minimum of 20-min in a supine position. Samples were centrifuged immediately after collection at 5,000 g for 10-min at 4°C, plasma was then carefully aliquoted to avoid disruption of the buffy coat, and frozen at −80°C until analysis. Plasma levels of oestradiol and progesterone were analysed by ELISA according to manufacturer’s instructions (ADI-900-008 and ADI-900-011, respectively, Enzo Life Sciences, Switzerland), with each sample run in duplicate on a single plate. All samples used in these analyses were subject to a single freeze-thaw cycle.

### 2.3 Data analysis

Data from 60 s of the baseline period, 30 s of the hypercapnic stages (from 2:30 to 3:00 min; Burley et al., 2020), and ≥20 s of data at the target end-tidal CO_2_ during the hypocapnic stages, were extracted and used in the statistical analyses. The linear slope of the cerebral blood velocity (CB*v*) response to changes in P_ET_CO_2_ was calculated to give an estimation of cerebrovascular-CO_2_ responsiveness in the MCA and PCA (MCA*v*-CO_2_ responsiveness; PCA*v*-CO_2_ responsiveness). Cerebrovascular responsiveness to hypercapnia, hypocapnia and hypo-to-hypercapnia were determined by the slope of the CB*v* response from stages Hyper1 to Hyper2, Hypo1 to Hypo2, and Hypo1 to Hyper1, respectively (Figure 1). The pulsatility index in the MCA and PCA (MCA*v*-PI; PCA*v*-PI) was calculated as (systolic CB*v*−diastolic CB*v*)/mean CB*v*.

### 2.4 Statistical analysis

Statistical analysis was performed using GraphPad Prism software (Version 8.0.0, GraphPad Software, United States). To compare outcomes measures in females across their menstrual cycle, a mixed effects model was used for all outcome measures to account for missing data (EF vs. O vs. ML). To compare males to females during menstrual phases of lower and higher oestrogen, two-way repeated measures ANOVAs were used for all outcome measures. Main and interaction effects of biological sex (males vs. females) and time [Visit A (early follicular phase, males trial 1) vs. Visit B (ovulatory phase, males trial 2)] were tested. Simple linear regressions were performed on cerebrovascular-CO_2_ responsiveness outcome measures to obtain a best-fit value of the CB*v* response across the P_ET_CO_2_ range (stages Hypo1, Hyper1 and Hyper; Figure 1). The line of best fit is reported as *y* = b*x* + a where b is the slope of the line and a is the *y*-intercept when *x* = 0. The slope and *y*-intercept values were compared between menstrual phases (EF vs. O vs. ML) and between males and females (males vs. EF vs. O). Data are presented as means ± SD. Statistical significance were based on an α-level of .05.

## 3 Results

Of the eleven female participants enrolled in the study, ten completed all experimental sessions, and one completed two sessions. However, with blood sample analysis of sex hormones, one participant was determined to not be in the ovulatory phase and therefore this visit was excluded from the analysis. Subsequently, the number of participants completing sessions in the EF, O and ML phases were 11, 9 and 11, respectively. All 10 males enrolled completed two sessions, however one male participant was excluded from the analysis due to a baseline P_ET_CO_2_ below that of the Hyper1 target P_ET_CO_2_.

### 3.1 Females across the menstrual cycle

#### 3.1.1 Plasma oestradiol and progesterone

A blood sample could not be obtained from one participant in their mid-luteal phase, and subsequently oestradiol and progesterone data are reported for 11, 9 and 10 participants in the EF, O and ML phases, respectively. On average, there was an increase in plasma oestradiol concentration during ovulatory (+60 ± 61%; range: 7%–170%) and mid-luteal (+10 ± 38%; range: −14% to 87%) phases compared to the early follicular phase, although this was not statistically significant (*p* = .08). Plasma progesterone concentration differed significantly across the menstrual phases (*p* < .01). Progesterone during the EF phase was significantly lower than both the O phase (−13% ± 19%; range: −42% to 18%; *p* < .05) and ML phase (−33% ± 21%; range: −69% to −6%; *p* < .01), and was greater during ML when compared to the O phase (+33% ± 34%; range: 4%–84%; *p* < .05).

#### 3.1.2 Baseline measures

Baseline responses can be found in Table 2. Heart rate, mean arterial pressure (MAP), ventilation and P_ET_CO_2_ were not significantly different between the EF, O and ML phases of the menstrual cycle (*p* ≥ .19). Further, MCA*v*, MCA*v*-PI, PCA*v*, and PCA*v*-PI at baseline were not significantly different between menstrual phases (*p* ≥.12).

**TABLE 2:** Baseline cardiovascular, respiratory, and cerebrovascular responses in females during three phases of their menstrual cycle, and in males during two repeat visits. Males are compared to females during the early follicular (EF) and ovulatory (O) phases only.

	Females, early follicular (*n* = 11)	Females, ovulatory (*n* = 9)	Females, mid-luteal (*n* = 11)	Males, trial 1 (*n* = 9)	Males, trial 2 (*n* = 9)
Heart Rate (bpm)	68 ± 7^a^	68 ± 7^a^ ^,^ ^b^	69 ± 10	62 ± 4	58 ± 6^b^
Mean arterial pressure (mmHg)	86 ± 8	85 ± 9	85 ± 7	86 ± 7	81 ± 7
Ventilation (L/min)	5.4 ± 1.3	5.5 ± 1.7	5.5 ± 1.1	6.6 ± 1.4	7.0 ± 2.5
End-Tidal CO_2_ (mmHg)	38.5 ± 2.5	38.9 ± 4.3	37.6 ± 3.7	38.6 ± 2.9	37.4 ± 2.7
MCA*v* (cm/s)	82 ± 17^a^	76 ± 17^a^	79 ± 15	66 ± 8	60 ± 8
PCA*v* (cm/s)	58 ± 8^a^	54 ± 13^a^ ^,^ ^b^	56 ± 12	47 ± 6	44 ± 7^b^
MCA*v*-PI (AU)	.68 ± .09	.77 ± .26	.71 ± .12	.83 ± .12	.83 ± .16
PCA*v*-PI (AU)	.69 ± .07	.74 ± .08	.72 ± .08	.85 ± .23	.81 ± .22

MCA*v*, middle cerebral artery velocity; PCA*v*, posterior cerebral artery velocity; MCA*v*-PI, middle cerebral artery pulsatility index; PCA*v*-PI, posterior cerebral artery pulsatility index. Values are means ± SD, n-1 for PCA outcome variables for male and female participants.

^a^ Main effect of biological sex, significantly different to males.

^b^ Main effect of time, significantly different to Visit A (females EF/males trial 1).

#### 3.1.3 Middle cerebral artery responsiveness

**FIGURE 2:**
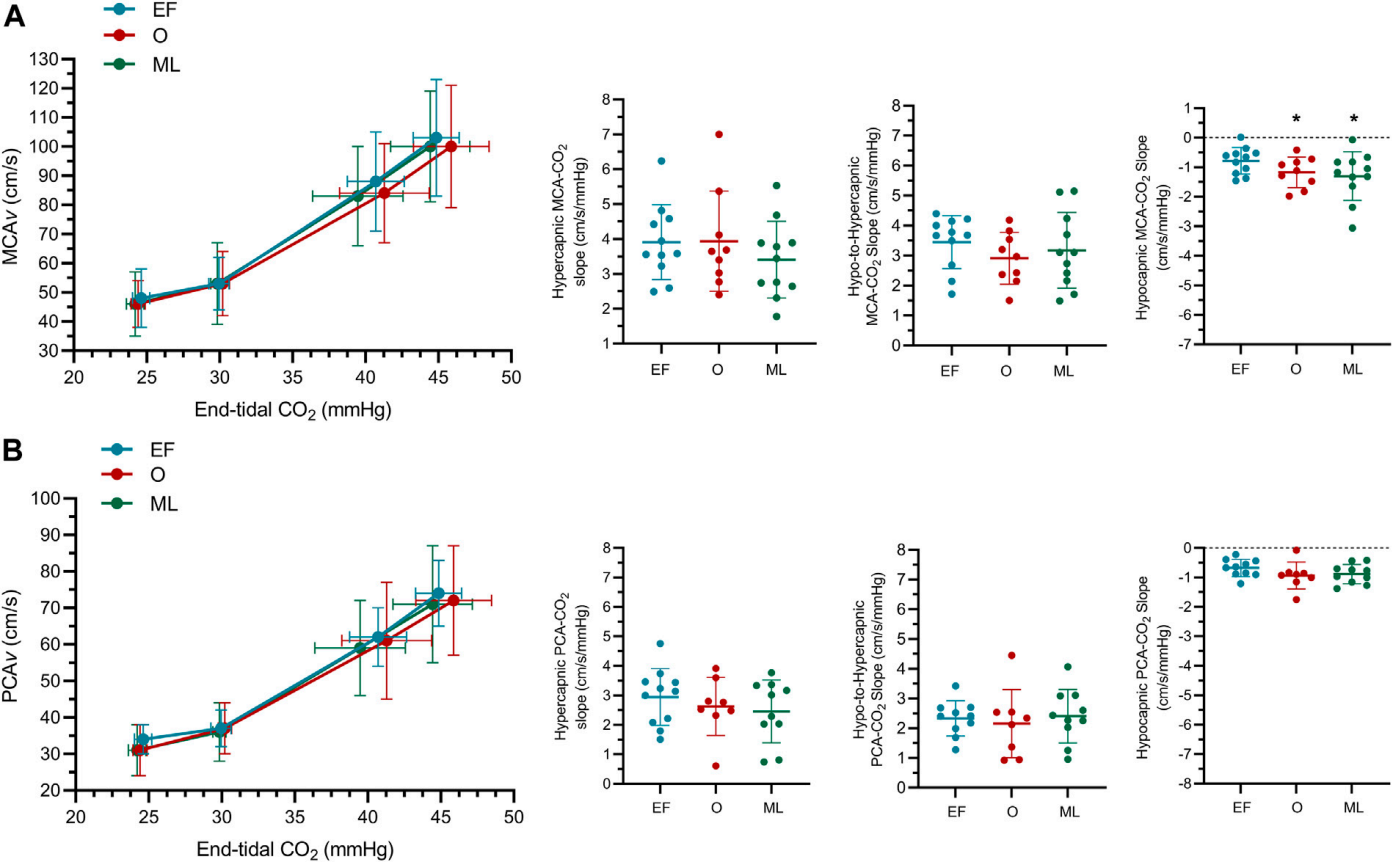
The middle **(A)** and posterior **(B)** cerebral blood velocity (MCA*v*; PCA*v*) slope at hyper-, hypo-to-hyper- and hypocapnia (MCA*v*-CO_2_ responsiveness, PCA*v*-CO_2_ responsiveness) for females during the early follicular (EF; *n* = 11), ovulatory (O; *n* = 9) and mid-luteal (ML; *n* = 11) phases of their menstrual cycle. n-1 for PCA outcomes. *significantly different to early follicular.

MCA*v*-CO_2_ responsiveness to hypocapnia was lower during EF when compared to the O phase (−.47 ± .48 cm/s/mmHg; *p* <.05; Figure 2; Table 3) and the ML phase (−.52 ± .70 cm/s/mmHg; *p* < .05). MCA*v*-CO_2_ responsiveness to hypocapnia was similar between O and ML phases (*p* = .79). MCA*v*-CO_2_ responsiveness to hypercapnia and hypo-to-hypercapnia was similar between menstrual phases (Figure 2; Table 3; *p* ≥ .25). Simple linear regression analysis of MCA*v*-CO_2_ responsiveness found similar slopes (*p* = .84) and *y*-intercepts (*p* = .57) between menstrual phases (EF, *y* = 3.39*x*−49.22; O, *y* = 3.02*x*−39.14; ML, *y* = 3.15*x*−40.75).

**TABLE 3:** Cerebrovascular-CO_2_ responsiveness values for the middle cerebral artery (MCA*v*-CO_2_ responsiveness) and posterior cerebral artery (PCA*v*-CO_2_ responsiveness) in females during three phases of their menstrual cycle, and males during two repeat visits (A, B). Males are compared to females during the early follicular (EF) and ovulatory (O) phases only. “Full range” refers to the linear regression line of best-fit which includes the Hypo1, Hyper1 and Hyper2 stages of the cerebrovascular responsiveness protocol.

		Females			Males	
		EF	O	ML	Trial 1	Trial 2
MCA*v*-CO_2_ responsiveness (cm/s/mmHg)	Hypercapnia	3.91 ± 1.07	3.94 ± 1.43	3.40 ± 1.10	3.15 ± .94	3.55 ± 1.96
	Hypocapnia	−.78 ± .45	−1.17 ± .52^a^	−1.30 ± .82^a^	−.78 ± .41	−.78 ± .40
	Hypo-to-hypercapnia	3.45 ± .88^b^	2.91 ± .87^b^ ^,^ ^c^	3.18 ± 1.26	2.40 ± .57	2.01 ± .46^c^
	Full range	3.39 ± .89	3.02 ± .85	3.15 ± .99	2.57 ± .56	2.39 ± .57
PCA*v*-CO_2_ responsiveness (cm/s/mmHg)	Hypercapnia	2.95 ± .96	2.63 ± .99	2.46 ± 1.01	2.49 ± .33	2.58 ± .65
	Hypocapnia	−.68 ± .29	−.94 ± .46	−.88 ± .33	−.50 ± .27	−.46 ± .28
	Hypo-to-hypercapnia	2.33 ± .59	2.15 ± 1.15	2.41 ± .91	1.67 ± .45	1.56 ± .40
	Full range	2.45 ± .54	2.25 ± .77	2.37 ± .75	1.83 ± .34	1.74 ± .44

Values are mean ± SD.

^a^ Significantly different to EF.

^b^ Main effect of biological sex, significantly different to males.

^c^ Main effect of time, significantly different to Visit A (females EF/males trial 1).

#### 3.1.4 Posterior cerebral artery responsiveness

PCA*v*-CO_2_ responsiveness to hypercapnia, hypocapnia and hypo-to-hypercapnia was similar between menstrual phases (Figure 2; Table 3; *p* ≥ .20). Simple linear regression analysis of PCA*v*-CO_2_ responsiveness found similar slopes (*p* = .91) and *y*-intercepts (*p* = .64) between menstrual phases (EF, *y* = 2.45*x*−36.55; O, *y* = 2.25*x*−31.74; ML, *y* = 2.37*x*−34.56).

### 3.2 Males vs. females (during EF and O phases)

#### 3.2.1 Baseline measures

Baseline responses can be found in Table 2. Heart rate was higher in females when compared to males (+9 ± 8 bpm; *sex: p* < .01). For both sexes, heart rate was lower during Visit B compared to Visit A (−3 ± 5 bpm; *time*: *p* = .01; *sex x time*: *p* = .28). MCA*v* was greater in females compared to males (+15 ± 16 cm/s; *sex*: *p* = .03) irrespective of menstrual phase (*time*: *p* = .11; *sex x time*: *p* = .95). PCA*v* was also higher in females compared to males (+11 ± 11 cm/s; *sex*: *p* = .02) and was lower during Visit B compared to the Visit A for both sexes (−5 ± 6 cm/s; *time*: *p* = .02; *sex x time*: *p* = .39). MAP, ventilation, P_ET_CO_2_, MCA*v*-PI, and PCA*v*-PI were similar between the sexes (*sex*: *p* ≥ .08) and between Visits A and B (*time*: *p* ≥ .38; *sex x time*: *p* ≥ .25).

#### 3.2.3 Middle cerebral artery responsiveness

**FIGURE 3:**
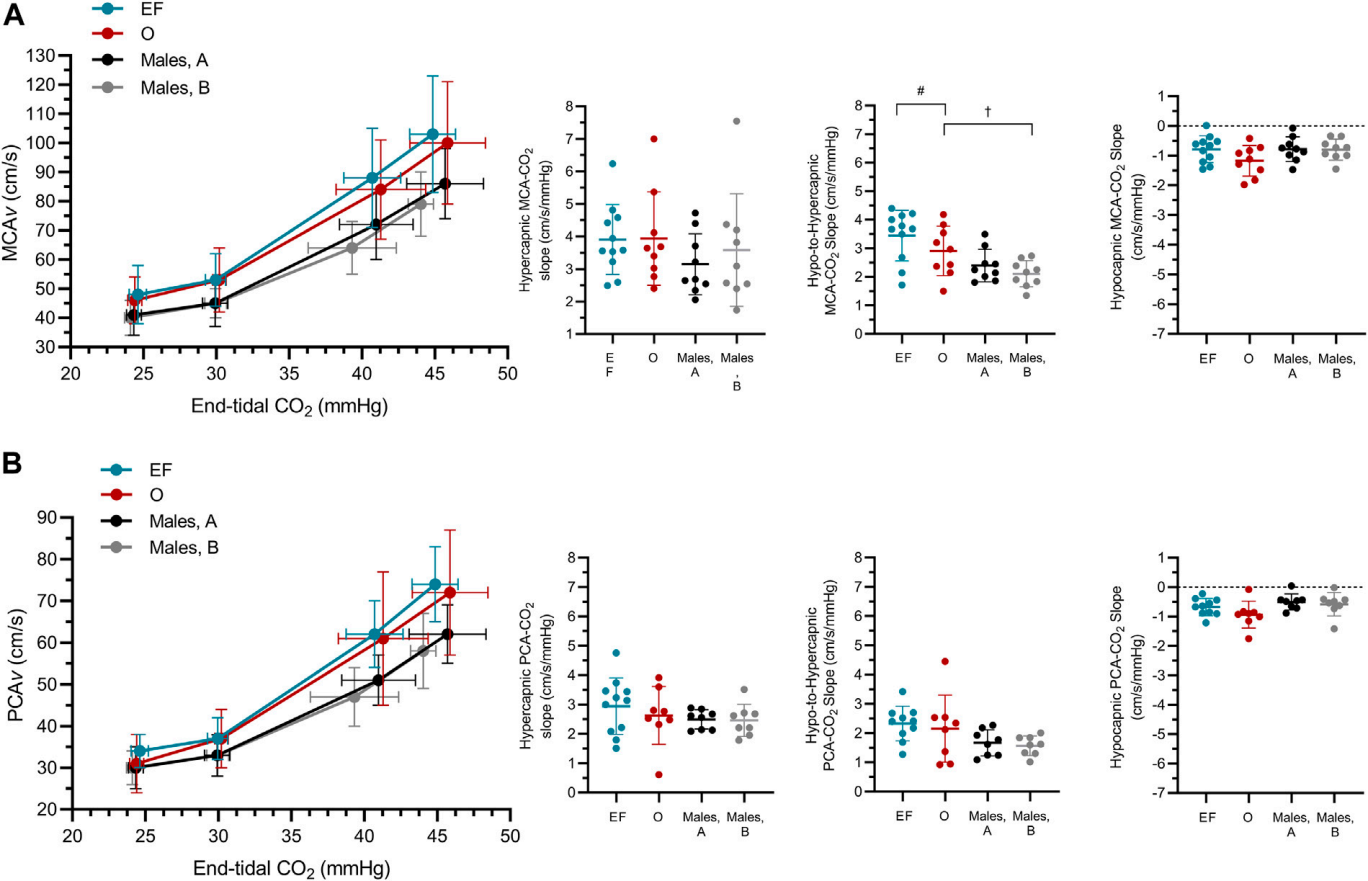
The middle **(A)** and posterior **(B)** cerebral blood velocity (MCA*v*; PCA*v*) slope at hyper-, hypo-to-hyper- and hypocapnia (MCA*v*-CO_2_ responsiveness, PCA*v*-CO_2_ responsiveness) in females during the early follicular (EF; *n* = 11) and ovulatory (O; *n* = 9) phases of their menstrual cycle, and in males (*n* = 9) during two repeated visits **(A,B)**. n-1 for PCA outcomes in both male and female participants. ^#^main effect of biological sex, significantly different to males; ^†^main effect of time, significantly different to Visit A (females EF/males trial 1).

MCA*v*-CO_2_ responsiveness to hypo-to-hypercapnia was higher in females compared to males (+.81 ± .97 cm/s/mmHg; *sex*: *p* = .03; Figure 3; Table 3) and was lower during Visit B compared to Visit A for both sexes (−.30 ± .59 cm/s/mmHg; *time*: *p* = .03; *sex x time*; *p* = .36; Figure 3; Table 3). MCA*v*-CO_2_ responsiveness to hypercapnia and hypocapnia were similar between the sexes (*sex*: *p* ≥ .31) and between visits (*time*: *p* ≥ .08; *sex x time*: *p* ≥ .09). Simple linear regression analysis of MCA*v*-CO2 responsiveness found similar slopes (*p* = .33) but differing *y*-intercepts (*p <* .01) between males and females (males A, *y* = 2.57*x*−32.26; males B, *y* = 2.39*x*−27.74; females EF, *y* = 3.39*x*−49.22; females O, *y* = 3.02*x*−39.14).

#### 3.2.3 Posterior cerebral artery responsiveness

PCA*v*-CO_2_ responsiveness to hypercapnia, hypocapnia and hypo-to-hypercapnia was similar between the sexes (*sex*: *p* ≥ .07), and between visits (*time*: *p* ≥ .26; *sex x time*: *p* ≥ .48). Simple linear regression analysis of PCA*v*-CO_2_ responsiveness found similar slopes (*p* = .24) but differing *y*-intercepts (*p* < .01) between males and females (males A, *y* = 1.83*x*−22.42; males B, *y* = 1.74*x*−19.54; females EF, *y* = 2.45*x*−36.55; females O, *y* = 2.25*x*−31.74).

## 4 Discussion

The main findings of this study were that 1) MCA*v*-CO_2_ responsiveness to hypocapnia was greater in females during O and ML compared to the EF phase of the menstrual cycle; 2) MCA*v*-CO_2_ responsiveness to hypo-to-hypercapnia was greater in females compared to males, regardless of which menstrual phase (EF or O) females were in. Finally, 3) PCA*v*-CO_2_ responsiveness across the CO_2_ range does not appear to differ across the menstrual cycle or differ between the sexes. Collectively, these findings indicate that females during the O and ML phases of the menstrual cycle have a greater vasoconstrictive capacity of the MCA compared to the EF phase, which we interpret as an improved functional capacity in the cerebrovasculature. Additionally, biological sex differences influence cerebrovascular-CO_2_ responsiveness in the MCA but not the PCA.

### 4.1 Effect of menstrual phase on cerebrovascular-CO_2_ responsiveness

The current study showed no difference in cerebrovascular-CO_2_ responsiveness to hypercapnia, but a greater MCA*v*-CO_2_ responsiveness to hypocapnia during O and ML phases as compared to the EF phase. Previous studies have reported that vasodilatory cerebrovascular responsiveness is both greater during O (Krejza et al., 2013) and similar between O and EF phases (Peltonen et al., 2016), although these studies used different vasoactive stimuli (acetazolamide and indomethacin, respectively) than used in the current study (CO_2_). Importantly, using a CO_2_ stimulus allowed us to examine both vasodilatory and vasoconstrictive responses of the cerebrovasculature. Subsequently, we found a greater vasoconstrictive capacity in the MCA during O and ML phases. Circulating oestrogen is associated with lower cerebrovascular tone (Krause et al., 2006) and therefore, greater resting cerebral blood flow in the internal carotid artery (Krejza et al., 2001) and the MCA (Peltonen et al., 2016) that may in turn allow for a greater vasoconstrictive capacity in response to hypocapnia. However, the present study found no difference in resting cerebral blood velocity or pulsatility index in the MCA between menstrual phases. Since TCD was used to insonate the vessels, changes in vessel diameter could not be measured, which would provide a more accurate indication of changes in flow *per se* and cerebrovascular tone. Regardless, at present, it appears unlikely that differences in baseline haemodynamics between menstrual phases cause the observed greater MCA*v*-CO_2_ responsiveness to hypocapnia in O and ML phases. Furthermore, in the present study oestrogen was not found to be significantly different between menstrual phases. Although this is likely due to high inter-individual variability in oestrogen responses (range of relative change from EF to O phase: 7%–170%), it cannot be established from our results whether changing oestrogen across the menstrual cycle causes the greater vasoconstrictive response in the MCA. Further investigation is warranted to determine the relationship between oestrogen and cerebrovascular-CO_2_ responsiveness, as well as the mechanisms by which oestrogen may cause a greater vasoconstrictive response to hypocapnia in the MCA.

We found a greater MCA*v*-CO_2_ responsiveness to hypocapnia during ML compared to the EF phase, and similar cerebrovascular-CO_2_ responsiveness between O and ML phases. To the best of our knowledge, no previous study has examined cerebrovascular-CO_2_ responsiveness to hypocapnia in the ML phase. However, the MCA*v* response to hypercapnia has been shown to be similar between EF and ML phases (Hazlett and Edgell, 2018), indicating progesterone may blunt the vasodilatory effect of oestrogen (Krause et al., 2006). The physiological pathways by which progesterone acts on the cerebrovasculature are less understood, with the majority of information arising from animal models. However, it is generally understood that progesterone acts as an antagonist to oestrogen, attenuating the anti-inflammatory response post-ischaemic brain injury (Sunday et al., 2006), and negating the vasodilatory effects of oestrogen in the peripheral vasculature (Miner et al., 2011). While caution needs to be taken when comparing the findings of the present study to such models, it may be expected that a rise in circulating progesterone during the ML phase would attenuate any effect elevated oestrogen levels may have on the cerebrovascular-CO_2_ responsiveness outcomes assessed here. However, our data indicates that the greater vasoconstrictive capacity seen during the O phase is maintained during the ML phase. Further research is needed to better understand the combined effects of changing oestrogen and progesterone levels throughout the menstrual cycle on cerebrovascular function, and the physiological pathways by which this occurs.

Cerebrovascular-CO_2_ responsiveness in the PCA remained similar between menstrual phases, indicating that MCA*v*- and PCA*v*-CO_2_ responsiveness to hypocapnia may respond independently across the menstrual cycle. Regional differences in cerebrovascular responsiveness have previously been reported, with the posterior circulation having a blunted hypercapnic response compared to the anterior circulation (Sato et al., 2012; Skow et al., 2013), while other findings suggest no differences in regional cerebrovascular-CO_2_ responsiveness (Willie et al., 2012). While these studies included females as part of their participant cohort, they either did not control for the menstrual cycle or included females in only the early follicular phase, making comparisons with the present study difficult. While the present study did not directly compare the MCA and PCA, the enhanced MCA*v*-CO_2_ responsiveness to hypocapnia in the O and ML phases indicates the MCA is more affected by menstrual phase than the PCA.

Reporting of oestrogen and progesterone has been previously recommended to provide confirmation of menstrual phase status, but also to help account for some of the variation in cerebrovascular outcomes between individuals (Skinner et al., 2021). The present study found changes in plasma oestradiol across the menstrual cycle to be highly varied between participants, with the relative increase in oestradiol from the early follicular to ovulatory phase ranging from 7% to 170%. Previously absolute levels of circulating oestrogen and progesterone throughout the menstrual cycle has been shown to be particularly varied between females (Windham et al., 2002). As such, the effects of sex hormones on the cerebrovasculature are likely to be highly individualised, occurring along a continuum rather than as definitive responses specific to certain phases of the menstrual cycle. Importantly, analysis of sex hormones showed that one participant was not in the ovulatory phase despite a positive ovulation test result, and data from this visit was subsequently removed from the analysis. Although this study recruited females with regular cycles, and used oral thermometers and ovulation kits, this illustrates that analysis of circulating sex hormones is still necessary to confirm a participant’s hormonal status.

### 4.2 Effect of sex on cerebrovascular-CO_2_ responsiveness

The present study found MCA*v*-CO_2_ responsiveness to hypercapnia and hypocapnia was similar between the sexes, however MCA*v*-CO_2_ responsiveness to hypo-to-hypercapnia was greater in females compared to males, irrespective of menstrual phase (i.e., EF vs. O). Further, PCA*v*-CO_2_ responsiveness was also shown to be similar between males and females across the CO_2_ range. Previous research investigating sex differences have shown cerebrovascular-CO_2_ responsiveness to be either greater in females compared to males (Kastrup et al., 1997; Oláh et al., 2000), similar between the sexes (Peltonen et al., 2015; Favre et al., 2020), or greater in males compared to females (Miller et al., 2019). Differences in the methods of previous studies (e.g., control of the menstrual cycle, method of cerebrovascular responsiveness examination) may account for the discrepancy in reported results. Additionally, previous studies have primarily insonated just the MCA and investigated the cerebrovascular vasodilatory response (i.e., hypercapnia), whereas the present study investigates both the MCA and PCA at four points across the CO_2_ range. While the present study did not formally compare the MCA*v*- and PCA*v*-CO_2_ responsiveness outcomes, observations indicate possible regional differences in cerebrovascular-CO_2_ responsiveness to hypo-to-hypocapnia between males and females, irrespective of menstrual phase. Subsequently, studies investigating cerebrovascular responsiveness to hypo-to-hypercapnia in the anterior circulation should consider the different responses exhibited by males and females.

In line with our hypothesis, the present study found MCA*v*-CO_2_ responsiveness to hypo-to-hypercapnia was greater in females compared to males. Circulating testosterone increases cerebrovascular tone (Gonzales et al., 2004), acting in an opposing manner to oestrogen. Subsequently, oestrogen and its activation of vasodilatory pathways (Krause et al., 2006) may cause a more dynamic MCA response to a hypo-to-hypercapnic perturbation. Conversely, males are likely to have a higher resting cerebrovascular tone due to greater circulating testosterone, possibly limiting their capacity to respond to changes in CO_2_ and explaining the lower cerebrovascular-CO_2_ responsiveness seen in males as compared to females in the present study. However, this is somewhat speculative, as sex hormone levels were not assessed in males and as previously discussed, cerebrovascular tone was not measured in the present study. While significant differences in resting MCA*v* and PCA*v* were observed between males and females, it is likely that this is primarily due to a smaller vessel diameter in females rather than hormonal differences between the sexes (Müller and Schimrigk, 1994). Similarly, sex differences in the MCA and PCA linear regression *y*-intercept most likely result from this known sex difference in the diameters of these vessels. Of note, both oestrogen and testosterone affect cerebrovascular tone in males and females (Krause et al., 2011). As such, it is likely the ratio of circulating oestrogen and testosterone in males and females influence observed differences in MCA*v*-CO_2_ responsiveness in the current study. However, sex hormones in males, and testosterone in females, were not examined in the present study and in future more robust reporting of hormones may be needed.

### 4.3 Considerations/study limitations

Cerebral blood velocity is generally reported to be higher in females compared to males and, as previously mentioned, is largely attributed to a smaller vessel diameter (Müller and Schimrigk, 1994). Accordingly, we found males to be significantly taller and heavier than females, as well as resting MCA*v* and PCA*v* to be significantly greater in females compared to males. To account for sex differences in cerebral blood velocity resulting from vessel diameter, relative changes in cerebrovascular responsiveness can be reported. We have reported the absolute change in cerebrovascular-CO_2_ responsiveness here as it is more pertinent to distinguishing clinically relevant changes in cerebrovascular function, as well as allowing for direct comparison to other populations. However, differences in physique between cohorts should be considered when interpreting the reported sex differences in cerebrovascular-CO_2_ responsiveness.

We used Doppler ultrasound to measure blood velocity in the MCA and PCA. A primary assumption of TCD is that the insonated vessel maintains a constant diameter. While this assumption has been validated (Valdueza et al., 1997; Serrador et al., 2000), more recent MRI studies have reported changing vessel diameters in response to changing CO_2_ (Coverdale et al., 2014; Verbree et al., 2014), which may result in possible over- and under-estimations of CBF when CBv is used as an index of absolute flow. Despite this, assessment of cerebrovascular responsiveness by TCD has been shown to offer valuable information on cerebrovascular health and function, provided data are interpreted with these limitations in mind (Willie et al., 2011).

In states of augmented hypo- or hypercapnia, the cerebrovasculature may reach a point of maximal constriction or dilation. The P_a_CO_2_ threshold for reaching the limit of the vessel calibre has been shown to be ∼65 mmHg in the hypercapnic range and ∼25 mmHg in the hypocapnic range (Harper and Glass, 1965). In the present study, P_ET_CO_2_ increased to ∼44 mmHg during Hyper2, and decreased to ∼24 mmHg during Hypo2. If the limit of the vessel calibre is reached, further diameter changes are not possible and a change in cerebrovascular-CO_2_ responsiveness may not be represented by further changes in CB*v*. To account for this, the Hypo2 stage was omitted from the simple linear regression, with analysis using the Hypo1, Hyper1 and Hyper2 stages.

The present study employed a steady-state CO_2_ technique to measure cerebrovascular-CO_2_ responsiveness. This technique was chosen as it incorporates both the ventilatory and cerebrovascular response to a steady-state CO_2_ stimulus. The use of different techniques to measure cerebrovascular- or ventilatory-CO_2_ responsiveness (i.e., steady-state or rebreathing) may elicit different outcomes as they stimulate physiological systems in a different manner. For example, a rebreathing technique abolishes the PCO_2_ gradient throughout the body (e.g., between end-tidal and arterial concentrations) and therefore, measures only the ventilatory response unaffected by the cerebrovascular response (Ainslie and Duffin, 2009). As such, the present findings should be considered with the methodology used in mind, and that outcomes may differ when reported alongside ventilatory responsiveness or if a different technique is used.

The present study did not control for several population demographics that may affect the reported outcome measures, including physical activity levels, sedentary behaviour, and training status. Greater aerobic fitness has previously been shown to be associated with lower baseline cerebral blood flow/velocity and greater cerebrovascular-CO_2_ responsiveness (Burley et al., 2017; Foster et al., 2020). Therefore, differences in fitness levels between males and females may contribute to some of the observed variation in cerebrovascular outcomes between cohorts.

While absolute values would ideally be used to assess menstrual cycle phase, relative values for oestradiol and progesterone are only reported in the present study due to high inter-plate variability within ELISA analyses. All samples for a single participant were run within a single plate allowing for reliable assessment of relative changes and menstrual phase confirmation.

An accurate sample size calculation could not be calculated *a priori* due to the current paucity of research in this area. Therefore, the small sample size of the present study should be considered when interpreting the results.

### 4.4 Summary

The findings of this study show that natural fluctuations in female sex hormones across the menstrual cycle influence cerebrovascular-CO_2_ responsiveness. A greater cerebrovascular-CO_2_ responsiveness to hypocapnia in the MCA during ovulatory and mid-luteal phases indicates an enhanced vasoconstrictive capacity during these phases. Further, biological sex differences in cerebrovascular-CO_2_ responsiveness are present irrespective of menstrual cycle. However, differences in cerebrovascular-CO_2_ responsiveness between the sexes and across the menstrual cycle are dependent on the insonated vessel, with the MCA appearing more responsive than the PCA.

## Data Availability

The raw data supporting the conclusion of this article will be made available by the authors, without undue reservation.
