# Supplementary material for: Menstrual phase influences cerebrovascular responsiveness in females but may not affect sex differences
Source: Front Physiol. 2023 Jan 4;13:1035452. doi: 10.3389/fphys.2022.1035452 (PMC9846518; doi:10.3389/fphys.2022.1035452)
Supplement: Supplementary file 1 [file Table1.DOCX]

Appendix 1 The number of female participants who completed the early follicular (EF), ovulatory (O) and mid-luteal (ML) phase as their first, second or third experimental visit. The order of visits was determined by which phase was identified first.

|  | **EF**  (n=11) | **O**  (n=10*) | **ML**  (n=11) |
| --- | --- | --- | --- |
| 1^st^ Visit | 7 | 2 | 2 |
| 2^nd^ Visit | 2 | 6 | 3 |
| 3^rd^ Visit | 2 | 2 | 6 |

*One participant’s ovulatory phase (their 2^nd^ Visit) was excluded after blood analysis of female sex hormones.
